# Biological ingredient complement chemical ingredient in the assessment of the quality of TCM preparations

**DOI:** 10.1038/s41598-019-42341-4

**Published:** 2019-04-10

**Authors:** Hong Bai, Xianhong Li, Hongjun Li, Jialiang Yang, Kang Ning

**Affiliations:** 1 School of Life Science and Technology, Huazhong University of Science and Technology, Wuhan, Hubei 430074 China; 2 College of Biological Sciences, Zhejiang University of Science and Technology, Hangzhou, 454000 P. R. China; 3 Department of Genetics and Genomic Sciences, Icahn School of Medicine at Mount Sinai, NewYork, USA

## Abstract

Traditional Chinese Medicine (TCM) preparations have been used in China for thousands of years. Quality evaluation for TCM preparations could be conducted based on chemical ingredients or biological ingredients. To date, the overwhelming majority of researches have focused on chemical ingredients while few studies were reported for biological ingredients. It is only recently that the assessments based on biological ingredients have drawn broader attentions. In this work, we have established a method for quality evaluation of TCM preparations by combination of chemical ingredients determined by HPLC fingerprint and biological ingredients obtained by high-throughput sequencing. This proof-of-concept method has been evaluated and compared with existing methods on Liuwei Dihuang Wan, a classical TCM preparation in China. By comparison of this method with those only based on chemical or biological ingredients, it is suggested that (1) Biological ingredient could complement chemical ingredient in separating TCM preparation from different manufacturers and batches with high accuracy; (2) classification of samples based on selected features would always out-perform those based on all features (either chemical or biological or both). By rationally selecting representative biological and chemical features, we have proven that these two types of features could complement each other for the assessment of ingredient consistencies and differences among various TCM samples, which is helpful to ensure the effectiveness, safety and legality of TCM preparations.

## Introduction

Traditional Chinese medicine (TCM) has been used in clinical applications for thousands of years in China, and it has drawn increasing attentions around the world recently due to its proven therapeutic effects with little side effects. Recently, crude materials and TCM extracts plays a dominant part in TCM export trade, while TCM preparations had weaker competitiveness^1,2^. One of the main reasons is that the quality and safety of TCM preparations remain key concerns around the world, which hinder their broader application and popularity among the international healthcare practitioners.

A TCM preparation is usually composed of several herbal materials with their respective dosages by the guidance of Chinese medicine theory, including pills, powders, capsules, tablets, and other dosage forms. One specified TCM preparation often contains a variety biological ingredients including plant, animal and mineral and exerts its therapeutic effects through the synergic effects of multiple active ingredients which involve mechanisms of multi-targets. Therefore, it is apparent that the quality of TCM preparations is important for their clinical efficacies. In 2015 Edition Chinese Pharmacopoeia (Ch. P.), quality evaluation of TCM preparations is mainly divided into the chemical ingredient (main chemical components) analysis and biological ingredient (species composition) analysis^3^. Methods for chemical ingredient analysis of TCM preparations include Thin Layer Chromatography (TLC) for qualitative analysis and High Performance Liquid Chromatography (HPLC) for quantitative analysis. It is worth mentioning that HPLC fingerprint technique, which could detect complex chemical constituents both qualitatively and quantitatively, plays an important role in samples’ differentiation and quality control of TCM preparations^4,5^. However, chemical ingredient analysis approaches could only determine compounds of interests, yet could not assess biological ingredients including prescribed species (species listed on the package) and unprescribed species (species not listed on the package) that actually were present in TCM preparations.

Recently, growing attentions have been paid on biological ingredient analysis for TCM preparations, especially for pill-based and powder-based preparations^6–9^. It is necessary to analyze the biological ingredients because of possible occurrences of incorrect identification, biological pollution, and adulteration during collection and production processes of herbal materials, which may affect the quality of TCM preparations and even cause serious illness for human beings^7^. The biological ingredient analysis methods for TCM preparations based on molecular diagnostic assay are widely used currently^10–12^, which have broken though the limitation of difficulties in discrimination of closely related species with similar morphology or containing the same chemical constituents.

The biological ingredient analysis for a TCM preparation could be abstracted as the process of identification of multiple species from a biological mixture. The metagenomic approach^13–15^ has been considered as one of the most effective methods for multiple species analysis from a biological mixture, which would also be helpful for the analysis of biological ingredients in TCM preparations. In previous studies, we have established a novel method (M-TCM) for biological ingredient analysis of a classical TCM preparation, Liuwei Dihuang Wan (LDW), based on metagenomic approaches via high-throughput sequencing (HTS), in which the ribosomal internal transcribed spacer 2 (ITS2) and the chloroplast genome *trnL* (UAA) intron were chosen as biomarkers^16,17^. Although M-TCM could assess the prescribed species and unprescribed species of LDW simultaneously, this method is still limited due to the nature of only using identifiable biological ingredients (that can be sequenced from TCM preparations) as features for differentiating TCM preparations.

Since chemical and biological ingredients are indivisible yet both important for quality assessments of TCM preparations, it would be natural to think about an integrated analytical method that could complement chemical ingredients by biological ingredients, which might provide a more accurate and general approach for the quality assessment of TCM preparations.

In this work, a proof-of-concept integrated method, which complement chemical ingredients with biological ingredients for TCM preparations, has been proposed. It was based on using the relative abundance of the selected chemical and biological features, and applying the random forest approach, for classification of TCM preparations.

As the object for exploration, LDW has been selected because it is a representative and widely used TCM preparation in China for the treatment of kidney Yin deficiency^18^, hypertension^19^ and osteoporosis^20^. LDW is composed of six herbal meterials, namely *Rehmannia glutinosa* Libosch., *Cornus officinalis* Sieb. et Zucc., *Paeonia suffruticosa* Andr., *Dioscorea opposita* Thunb., *Poria cocos* (Schw.) Wolf, and *Alisma orientalis* (Sam.) Juzep. In the previous studies on LDW, chemical ingredients were analyzed by HPLC^21–23^ and biological ingredients were determined based on HTS^17^. In the present study, as the biological ingredient data for LDW has already been obtained from previous work^17^, the chemical fingerprint of LDW was collected on the same batch of samples.

Since we have no standard substances of LDW, the random forest approach^24^ has been applied to classify samples based on their profiles generated by the integration approach, followed by accuracy evaluation of sample classification by comparison with ground-truth about manufacturers and production batches of the LDW sample, as well as compared with the classification results only based on chemical or biological ingredient information. We have also benchmarked the random forest approach by comparison with results of K-nearest neighbor (KNN) approach.

Results have shown that the integrated approach could successfully differentiate TCM preparation from different manufacturers and batches with high accuracy, and classification of samples based on selected features would always out-perform those based on all features (either chemical or biological or both). Thus, this integrated approach that complement chemical and biological ingredients, represents a solid step towards a more accurate and holistic assessment of TCM preparations.

## Methods

### Sample preparations

#### Samples and reagents

9 commercial LDW specimens were purchased from 3 different Chinese manufacturers (namely MH, MS and MT) and each with 3 batch numbers (A, B and C) (Supplementary Table S1). Each batch was implemented with 3 parallel repeats, therefore there were totally 3 × 3 × 3 = 27 commercial LDW samples. HPLC-grade methanol and acetonitrile were purchased from Tedia Company (USA). Ultra-pure water was prepared by using a Milli-Q50 SP Reagent Water System. Phosphoric acid (HPLC-grade) was purchased from Sinopharm Chemical Reagent Co. Ltd. (China).

#### Instrumentation and chromatographic conditions

The HPLC system was comprised of a Waters 1525 analytical HPLC equipped with a DAD detector. An Agilent SB-C_18_ column (4.6 mm × 250 mm, 5 μm) was used for separation, and the column temperature was 30 °C. The mobile phase was consisted of acetonitrile (A) and water (B, with 0.1% phosphoric acid) with the following gradient program: 0–15 min, 1–12% A; 15–45 min, 12–50% A; 45–55 min, 50% A; 55–55.1 min, 1% A; 55.1–65 min, 1% A. The flow rate was set at 1 mL/min, and the DAD wavelength was 236 nm, with a sample injection volume of 10 μL.

#### Sample preparation for chemical assessment method

A LDW sample (3.0 g) was cut into small pieces and ground with 5 mL distilled water to get a homogeneous solution. Single extraction for each sample was performed by precise weighing 3.0 g of homogeneous solution above, and then placing it in a flask together with 20 mL methanol. Then, the mixture was extracted by ultrasonication for 30 min and then allowed to stand for 60 min at room temperature to cool down. The lost weight of the mixture was supplied by methanol. The extraction solution was subsequently filtered through a 0.45 μm nylon membrane filter into a HPLC vial prior to analysis.

#### Method validation for HPLC fingerprint analysis

The precision and repeatability of the HPLC fingerprint method, as well as the stability of the samples was assessed by the relative standard deviation (RSD) of retention time (RT) and peak area (PA). The precision was expressed as the RSDs of analyzing the same LDW extract solution five times. The repeatability was assessed by analyzing five extract solutions prepared independently of LDW samples. To investigate the stability of the analytical framework, the extract solution was analyzed after being stored at room temperature for 0, 5, 10, 20, and 40 hours, respectively.

#### Sample preparation and analyses for biological ingredient

The steps of DNA extraction, amplification, sequencing and data analysis were described in our previous work^16,17^. Briefly, DNA was extracted by TCM-CTAB method, and these DNA extracts were amplified by traditional PCR (by using ITS2 as biomarker) before sent for 454 sequencing. The 454 sequencing data of 27 LDW samples was deposited and could be obtained from NCBI SRA database with accession number SRR1049940. Based on 454 sequencing data, quality control, species identification and reads mapping of each species have been performed by standard method^17^ for each LDW sample.

### Similarity analysis based on chemical ingredients

LDW samples were analyzed by the established HPLC method above. The HPLC data obtained of 27 samples were exported from Waters Empower 2 software in AIA format and imported to the professional software named *Similarity Evaluation System for Chromatographic Fingerprint of Traditional Chinese Medicine* (Version 2004A) with default parameters. This software could reflect the similarity of distribution ratio of the chemical compositions, which was recommended by China’s State Food and Drug Administration (SFDA). A simulative Mean Chromatographic Fingerprint (MCF) was calculated and used as a representative fingerprint.

### Similarity analysis based on biological ingredients

The relative abundance of each detectable species in 27 LDW samples was generated by the Parallel-META software^25^, which was used for similarity analysis among different LDW samples based on biological ingredients. In terms of the similarity calculation method, a similarity value between every two samples was computed by “1 - Euclidean distance”, in which the Euclidean distance^26^ was calculated based on the relative abundance of detectable species of the two samples compared.

### Hierarchical clustering analysis

For clustering of the LDW samples, average-linkage hierarchical clustering algorithm by the “hclust()” function of R^27^ was applied to cluster LDW samples based on their similarities of chemical ingredients and of biological ingredients, respectively. A heat map figure was generated based on the clustering result by the “gplots” package of R (http://CRAN.R-project.org/package=gplots, R package version 2.12.1 (2013)).

### Strategy for chemical and biological feature selection

For chemical data from HPLC, each peak was regarded as a feature while for biological ingredient data, each species was considered as a feature. There were 55 chemical features (Supplementary Table S2) and 35 biological features (Supplementary Table S3) for 27 LDW samples.

For the selection of features, first, all the features (55 chemical features and 35 biological features) were taken as training datasets with random forest algorithm and then each feature’s importance score was calculated through permuting values of this feature and then calculating and normalizing the difference of out-of-bag errors before and after a permutation^24^. Then features were added one by one according to importance score of the feature (with descending order). 10-fold cross validation for 500 times for each type data was performed until the mean prediction accuracy reaching the optimal value.

### Strategy for integration of chemical and biological ingredients

As the results of chemical and biological ingredient information were largely different not only in their targets but also in their data format, such heterogeneity placed a significant hurdle to this integration approach. However, as these two types of information had a common property that each feature could be described by its relative abundance, an integrated approach was proposed based on the combination of relative abundance of chemical and biological features. The approach was consisting of two steps: firstly, selection of features from both chemical and biological profiles according to the feature importance score computed by random forest, and secondly, combination of these features normalized through their relative abundance, as a set of integrated features.

### Strategy for assessment of the classification performance

For data of each type (all chemical features, all biological features, selected chemical features, selected biological features and integrated features), a random forest method was applied on different sets of training and testing data for classification of LDW samples, and these classification results were then compared against the ground-truth about LDW sample manufacturers and production batches for accuracy assessments. With details as follow: Firstly, all 27 LDW samples were divided randomly into 10 groups, in which 9 groups as training data and 1 group as testing data. Secondly, 500 bootstrapping steps were repeated and the average accuracy value of each set were calculated and evaluated. K-Nearest Neighbor (KNN) method has also been conducted on the same sets of training/testing datasets, fo comparison with the random forest method. ROC (Receiver Operating Characteristic) curves results were plotted manually by the true positive rate (TPR) against the false positive rate (FPR) based on the results for various training/testing sample settings.

## Results

### Consistency analysis of LDW samples from different manufacturers based on chemical ingredients

#### Optimization of HPLC conditions

In order to obtain a HPLC chromatogram with good separation of adjacent peaks within reasonably analysis time, different mobile-phase compositions with methanol and acetonitrile were investigated. It was found that a linear gradient elution with acetonitrile and 0.1% aqueous phosphoric acid was the most suitable eluting solvent system. The DAD detector was employed at the wavelength ranging from 190 nm to 400 nm for obtaining a sufficient number of detectable peaks. As a result, 236 nm was selected by consideration of the number of detectable peaks and the resolution.

#### Assessment of the validity of HPLC fingerprint analysis method

To ensure the validity of HPLC fingerprint analysis method, sample MT.A was randomly selected for precision, repeatability and stability tests. Intraday precision and repeatability as well as interday stability of the method were determined and expressed by the relative standard deviation (RSD) values of the retention time (RT) and peak area (PA) of six characteristic peaks, summation of whose PAs were about 60% of the total PA (Table 1). All validation tests had proven that the HPLC method was reliable.

**Table 1:** Analytical results of the precision, stability and repeatability for 6 characteristic common peaks in LDW sample (MT.A) (n = 5).

No.	Mean RT (Min)	RSD of RT (%)			RSD of PA (%)		
		Precision	Repeatability	Stability	Precision	Repeatability	Stability
1	24.55	0.89	0.63	0.21	1.17	0.32	0.67
2	27.90	0.78	0.72	0.19	0.58	0.55	0.62
3	30.22	0.79	0.73	0.21	2.67	2.79	0.22
4	36.10	0.70	0.67	0.21	1.95	1.73	0.27
5	42.59	0.62	0.60	0.12	0.34	0.75	0.40
6	48.22	0.24	0.24	0.13	0.34	0.79	0.28

#### Similarity analysis based on HPLC fingerprint of 27 LDW samples

**Figure 1:**
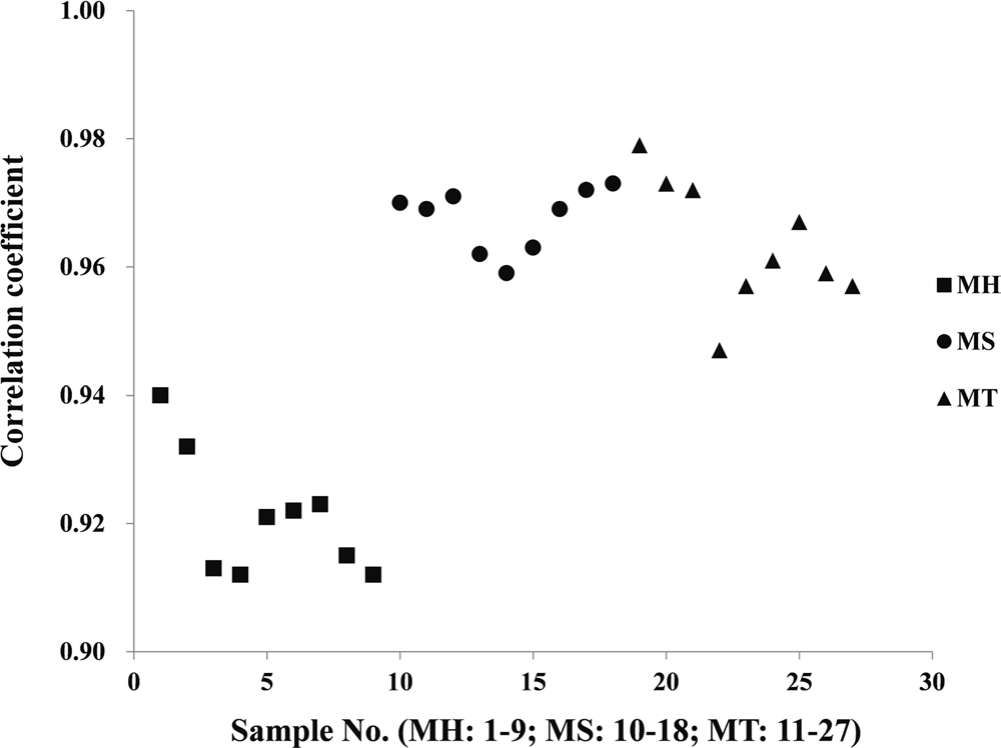
The correlation coefficients of LDW samples from three manufacturers (MH, MS and MT) compared to Mean Chromatographic Fingerprint.

**Figure 2:**
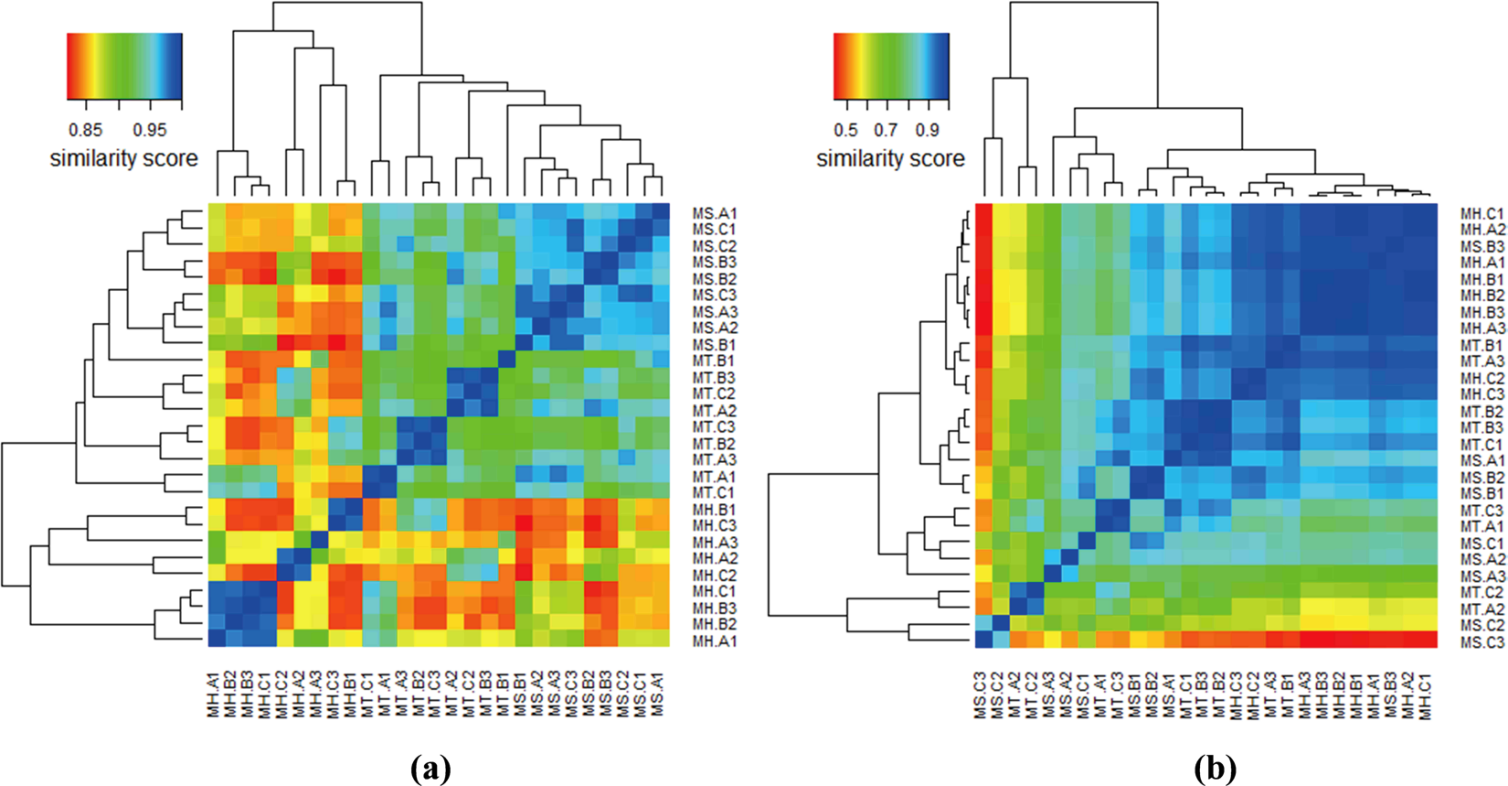
Heat maps showing clusters of 27 LDW samples using hierarchical clustering based on (**a**) HPLC fingerprint similarity and (**b**) detectable species similarity.

Consistency of chemical ingredients is an important consideration in quality evaluation of TCM preparations. HPLC fingerprint chromatograms of 27 LDW samples (Supplementary Table S2) were shown in Supplementary Fig. S1(a). It was obviously that there was a random error for sample MT.B2 (S23) since baseline shift was observed at 60.315 min. In order to eliminate the accidental error above, the analysis time for one round was trimmed from 65 min to 60 min due to the absence of the peak after 60 min (Supplementary Fig. S1(b)). The similarities between different pairs of LDW samples based on HPLC fingerprint were calculated by *Similarity Evaluation System for Chromatographic Fingerprint of Traditional Chinese Medicine* (Version 2004A) and expressed by correlation coefficient values (Supplementary Table S4). The value of the correlation coefficients closer to 1.0 indicated the higher degree of similarity of two samples. Figure 1 showed the distribution of correlation coefficients derived from different samples, which made it clear that the samples from MH had relatively low similarity (0.91–0.94) to Mean Chromatographic Fingerprint (MCF) while the samples from MS and MT exhibited higher similarity (0.95–0.98) to MCF. Meanwhile, the small variation of batches’ similarity was observed from MS manufacturer. To further explore the differences among 27 commercial LDW samples, a clustering analysis using average-linkage hierarchical clustering algorithm based on the similarity was performed. Results shown in the heat map (Fig. 2a) were largely consistent with the results shown in Fig. 1, namely, the samples from MS and MT clustered together while MH samples showed larger inter-group differences. These results indicated that the degree of consistencies for chemical ingredients were different among samples in different manufacturers and batches.

In order to discover major factors that influence the variation of the different samples, RT and PAs of 27 LDW samples were analyzed. 55 Peaks were observed with RSD of RT less than 1.18%, among them 11 peaks occurred in all samples (Supplementary Table S2). Except for 11 common peaks, the appearance time of other peaks changed irregularly, which resulted in that RSD of PA for 55 peaks varied significantly from 0% to 81.8%. By careful comparison of the RT and PA data in Supplementary Table S2, it was found that MH samples were largely different from MT and MS samples mainly due to the fact that the PAs of some common peaks were far away from that of MT and MS, such as PAs of the peaks at 24.49, 27.79, 29.34, and 30.10 min. In addition, for MH samples, an individual peak at 39.28 min and the absence of the peak at 23.76 min were also the influencing factors for their differences from that of MT and MS.

### Consistency analysis of LDW samples from different manufacturers based on biological ingredients

#### Selection of biomarkers

In our previous work, the biological ingredients of 27 LDW from three manufacturers based on high throughput sequencing by using ITS2 and *trnL* as biomarkers^17^ were reported. Although ITS2 and *trnL* could both be used as biological ingredient analysis, in this study ITS2 was chosen as biomarker due to its broader application and higher sensitivity.

#### Similarity analysis based on detectable species of 27 LDW samples

In total, 35 detectable species were found based on ITS2 of 27 LDW samples. To be consistent with the similarity assessment based on chemical ingredients, the relative abundance of each detectable species (Supplementary Table S3) was used for the similarity analysis among different LDW samples based on biological ingredients. The similarity value was calculated by “1 - Euclidean distance (Supplementary Table S5)” for each two samples. The value of similarity closer to 1 indicated higher degree of similarity of the two samples, while that closer to 0 indicated higher degree of difference. From the heat map (Fig. 2b), It could be observed that MS.C2 and MS.C3 were significantly different form the other samples. Further data analysis indicated that this difference might be due to lower abundance of a prescribed species *Paeonia suffruticosa* while higher abundance of a species from *Vigna* genus, which was assigned to unprescribed species. In addition, two samples form MT (MT.A2 and MT.C2) were also distinctly different from the other samples due to higher abundance of a prescribed species *Alisma orientalis* and lower abundance of a prescribed species *Paeonia suffruticosa*.

Sample clustering results were obviously different between Fig. 2a,b. Samples clustered by manufacturers were shown and the difference among batches was small in Fig. 2a while the variation among manufacturers and batches were evident in Fig. 2b. In addition, the cluster degree of MH was better than that of MS and MT in Fig. 2b. Since chemical features and biological features were different assessment indices aimed at different targets during TCM quality evaluation, which could give distinct but complementary evaluation results.

### Consistency analysis of LDW samples based on complementation of chemical and biological ingredients

From chemical ingredient and biological ingredient analysis of LDW samples above, it was observed that results based on chemical ingredient analysis would provide clearer separation of samples according to manufacturers than results based on biological ingredient analysis. However, chemical evaluation cannot discover impurity ingredients and cannot reflect the species information in the TCM preparation, thus it still mis-classify some of the samples to wrong manufacturers in certain runs. The integration approach that complements chemical ingredients with biological ingredients might further improve accuracy for differentiating samples according to their manufacturers or batches.

#### Selection of chemical features and biological features

**Figure 3:**
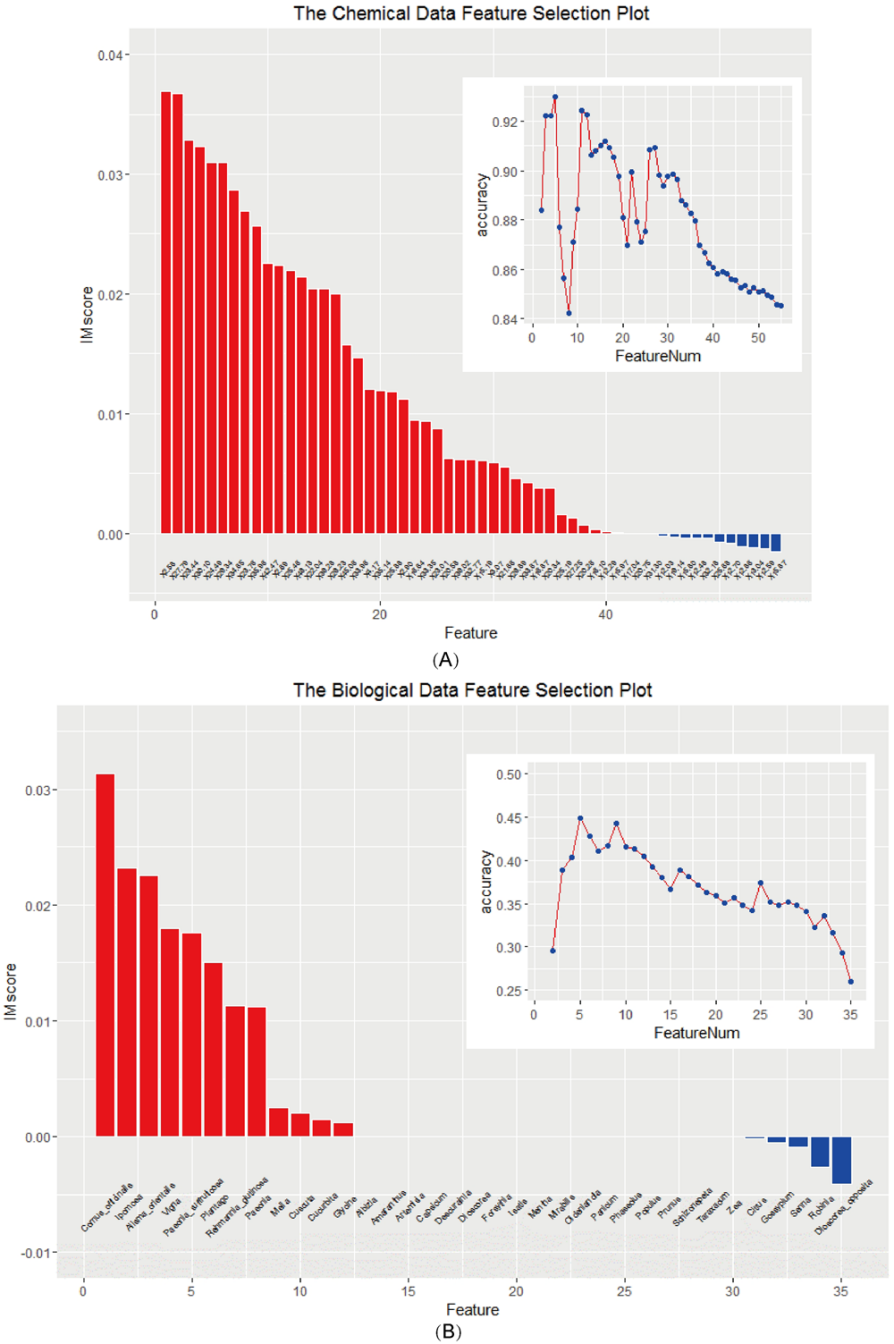
Feature selection results. The main figure represented the rank of each (**A**) chemical ingredient’s and (**B**) biological ingredient’s importance score (IM score) calculated by random forest algorithm and the top 5 ingredients were selected as features. The sub-figure represented the change of mean prediction accuracy of 10-fold cross validation 500 times for each combination of (**A**) chemical ingredients and (**B**) biological ingredients added one by one according to importance score of the ingredient (both starting with combination of the first two ingredients).

Based on pre-defined batch group information (small cluster), namely to select features that would best differentiate samples among different batches by random forest algorithm, 5 peaks at 2.58, 27.79, 23.44, 30.10 and 24.49 min and 5 species including *Cornus officinalis*, *Ipomoea*, *Alisma orientalis*, *Vigna* and *Paeonia suffruticosa* (ordered by their importance score shown as Fig. 3A,B) were selected.

Considering the influence of background noise and processing time used in full chemical or biological features, as well as the relative lower accuracy value in random forest test (sub-figures in Fig. 3A,B), it was learned that the selected features would also have advantages compared with full sets of chemical or biological features. Thus, only selected chemical and biological features were used for the assessment of integrated features.

In the following analyses, these selected features were utilized as follow: the normalized intensities of 5 selected chemical features (peaks at 2.58, 27.79, 23.44, 30.10 and 24.49 min) and 5 selected biological features (*Cornus officinalis*, *Ipomoea*, *Alisma orientalis*, *Vigna* and *Paeonia suffruticosa*) were combined directly as the integrated feature. This is due to the fact that these two types of features are of different properties and are from different experiments on the same samples.

#### Evaluation of classification performance of the features selected

**Figure 4:**
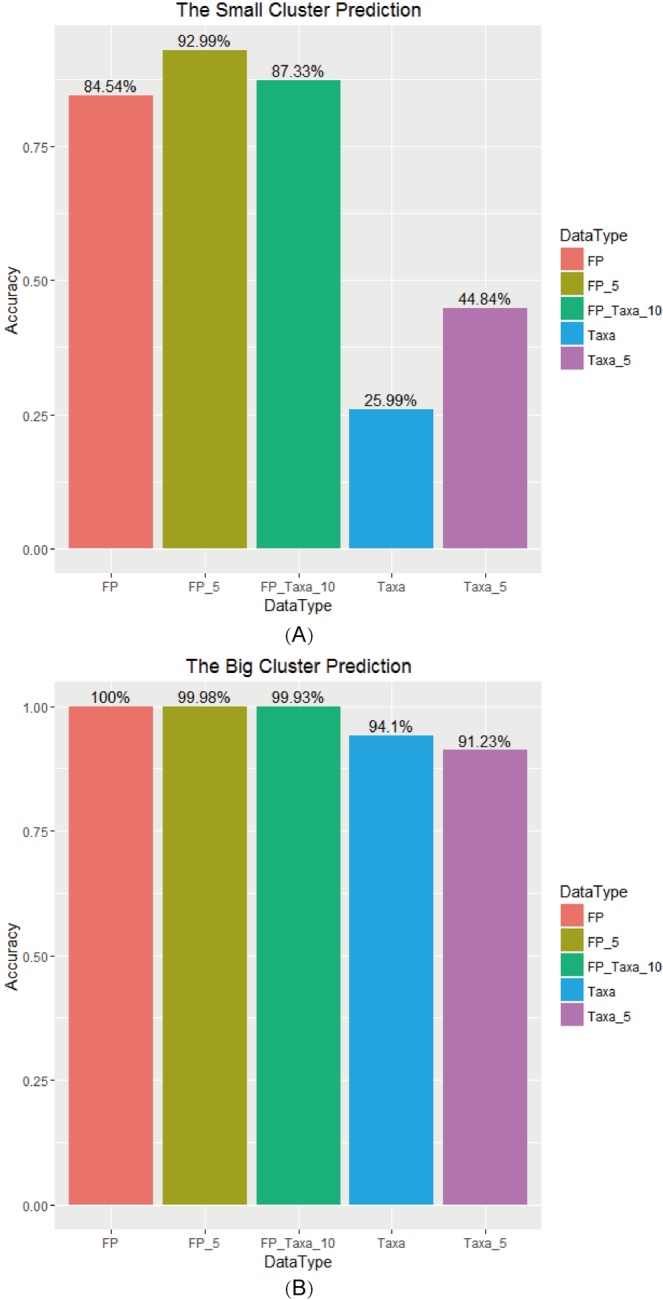
Accuracy analysis of different LDW samples based on random forest method. Bar plot of accuracy value based on chemical and biological ingredients in differentiation of samples among (**A**) different batches of different manufacturers and (**B**) different manufacturers. Category information: FP, all chemical features; FP-5, 5 selected chemical features; Taxa, all biological features; Taxa-5, 5 selected biological features; FP_Taxa_10, integrated features.

For chemical features, clustering analysis might be hampered by the existence of noises within detectable peaks, so the accuracies of sample classification based on two settings, 5 selected chemical features and all chemical features, were assessed by random forest with 500 bootstrapping repeats. Mean accuracy values were obtained from training/testing sample settings (each batch of a specific manufacturer was considered as a cluster, small cluster) (Fig. 4A). It was clear that the classification accuracy based on 5 selected chemical features (FP_5) was near 93%, and the result on all peaks (FP) was less accurate than those based on selected peaks, indicating that there might exist unneglectable noises in all detectable constituents.

In order to evaluate the performance of chemical ingredients on the manufacturer class (each manufacturer was considered as a cluster, big cluster), selected chemical features (FP_5) and all chemical features (FP) was also used to distinguish samples from different manufacturers (Fig. 4B). The classification accuracies based on FP_5 and FP were both extremely high with slightly difference. According to above results, it was obvious that the result based on FP-5 clearly showed the best accuracy among FP in small cluster classification.

Similarly, in the investigation of the effect of selected biological features on sample classification, we have also observed the advantage of using selected features. From Fig. 4A, we could see that the classification accuracy in small cluster based on 5 selected features (Taxa_5) better than the result based on all species (Taxa). This also suggested that there might be unneglectable noises in all detectable species.

#### Comparison with integrated methods

For the integrated features, namely, the combination of the normalized intensities of 5 selected chemical features and 5 selected biological features (FP_Taxa_10). From Fig. 4A, it could be observed that the accuracy of the classification based on integration features (FP_Taxa_10) was placed between those based on FP features and on FP_5 features and far better than the Taxa features (Taxa and Taxa_5). While classification accuracy based on Taxa features was much lower from those based on integrated features and FP features. From Fig. 4B, the accuracies based on FP_Taxa_10 in differentiation of samples among different manufacturers were similar with those based on all chemical features (FP), and higher than those based on biological features (Taxa) in general (near 100% for differentiation of samples from different manufacturers).

According to the above results, the accuracy of FP_Taxa_10 in differentiation of samples among different manufacturers and samples among different batches (average 99.93% and 87.33%, respectively) were high enough, and the effects were stable for performing the classification among all types of features. These performances was not as good as the best types of features (FP_5 and FP, respectively), but only with a small gap for batch-level and manufacturer-level predictions (Fig. 4A,B).

### Benchmarking based on KNN approach

**Figure 5:**
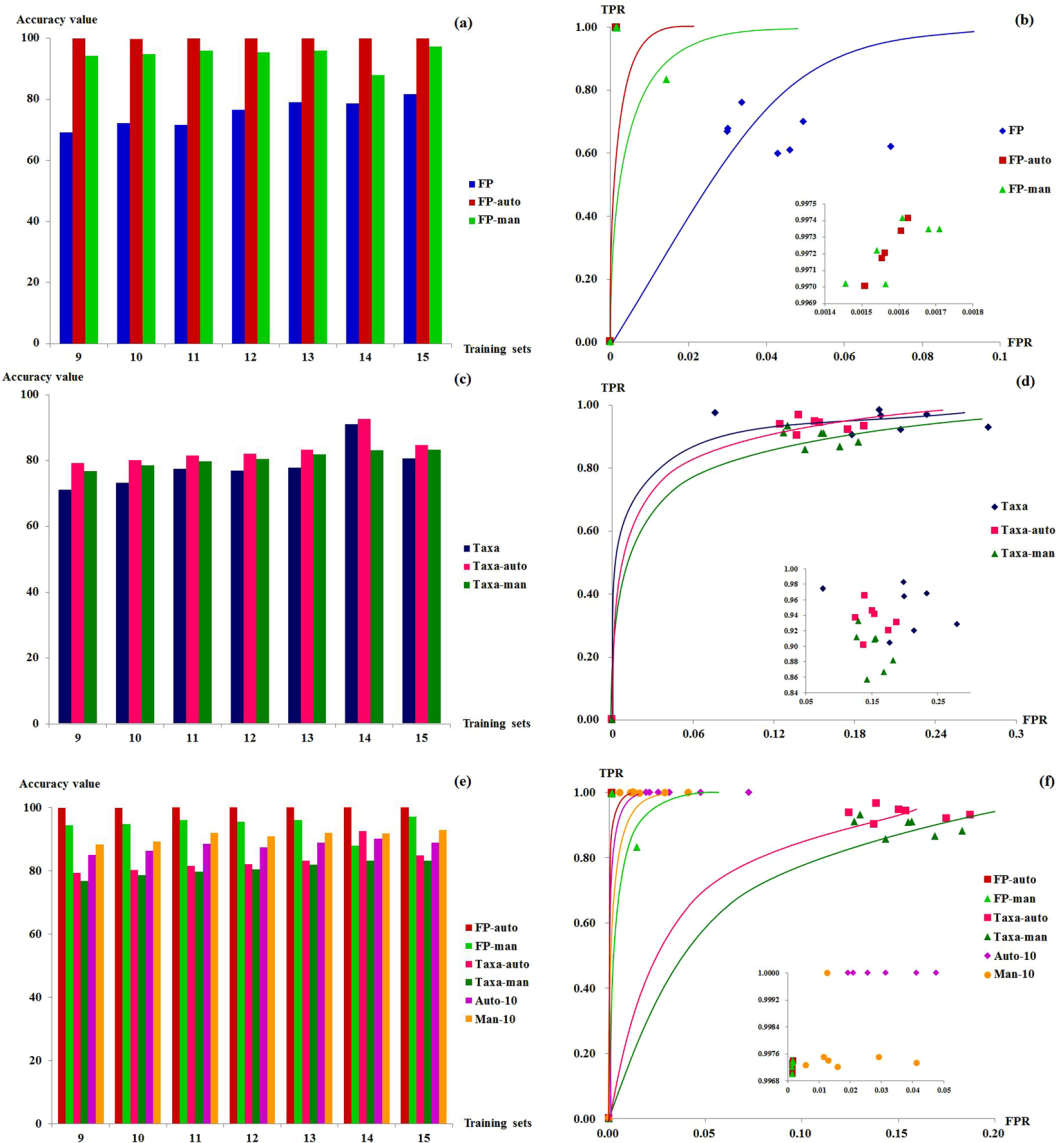
Accuracy analysis of different LDW samples based on KNN method. (**a**) Bar plot of accuracy value based on chemical ingredients; (**b**) ROC curve based on chemical ingredients; (**c**) Bar plot of accuracy value based on biological ingredients; (**d**) ROC curve based on biological ingredients; (**e**) Bar plot of accuracy value based on both chemical and biological ingredients; (**f**) ROC curve based on both chemical and biological ingredients. Insets for (**b**,**d**,**e**): enlarged ROC figures at top-left that could show differences among different approaches. For Fig. 3a,c,e, X-axis represents the number of samples used for the training set, and Y-axis represents accuracy value for classification. Here accuracy value refers to the classification accuracy for samples in testing set at the manufacture level (not batch level). (2) For Fig. 3b,d,f, X-axis represents false positive rate (FPR) for classification, and Y-axis represents true positive rate (TPR) for classification. (3) Category information: FP, all chemical features; FP-auto, 5 automatically selected chemical features; FP-man, 5 manually selected chemical features; Taxa, all biological features; Taxa-auto, 5 automatically selected biological features; Taxa-man, 5 manually selected biological features; Auto-10, 5 automatically selected chemical features and 5 automatically selected biological features, Man-10: 5 manually selected chemical features and 5 manually selected biological features.

To verify the results based on random forest for complementing chemical features and biological features for TCM sample differentiation, we have also applied KNN on the same sets of training/testing datasets. The sample classification accuracies based on three settings, 5 manually selected chemical features, 5 automatically selected features and all chemical features, were assessed by KNN with 1,000 bootstrapping repeats. Seven sets of accuracy values were obtained from 7 training/testing sample settings (Fig. 5a). It was shown that classification accuracies based on 5 automatically selected features (FP-auto) were near 100% and always more accuracy than those based on 5 manually selected features (FP-man), probably due to the fact that manually selected features only contains peaks with bigger PA and ignores chemical constituents with lower abundances. Moreover, results on all peaks (FP) would always be less accurate than those based on selected peaks, indicating that there might exist non-neglectable noises in all detectable constituents.

In order to visualize and compare KNN classification results based on all and selected chemical features, ROC (Receiver Operating Characteristic) curves for FP, FP-auto and FP-man were plotted manually by the true positive rate (TPR) against the false positive rate (FPR) based on the results for various training/testing sample settings (Fig. 5b). It was obvious that the results based on FP-auto clearly showed the best accuracies among FP, FP-auto, and FP-man.

Results on selected biological features (Taxa-auto and Taxa-man) have shown similar ROC compared to the results based on all taxonomical features (Fig. 5c,d). And for integrated approach, we have observed that results based on automatically selected features have the best performances (Fig. 5e,f).

Based on this benchmarking experiments by KNN, we have confirmed the two key findings of this work: (1) Biological ingredient could complement chemical ingredient in separating TCM preparation from different manufacturers and batches with high accuracy; (2) classification of samples based on selected features would always out-perform or are comparable with those based on all features (either chemical or biological or both features).

## Discussions

By complementing chemical ingredients with biological ingredients, we have observed that such integrated approach could outperform that based on chemical ingredient only approach, yet this is true just for the majority of samples but not so for other samples. This is true based on either using random forest or KNN classifier as the method to differenciating samples. Careful analyses of the results indicated that the reason might be that the classification accuracies based on selected chemical features was much better than those based on selected biological features. For quality evaluation of TCM preparations, methods based on chemical ingredients have been prevailed several decades while methods based on biological ingredients have just emerged in recent years. In this study, the relative abundances of chemical constituents were given by the high quality software already recommended for quality evaluation of TCM preparations by SFDA, while based on newly emergent analytical package, estimation of the relative abundance of detectable species still had large room for improvement. Thus, it is nature that the results based on chemical method were better than that based on biological method as of now. However, it was still true that biological ingredient could complement chemical ingredient for classification of TCM preparations, according to manufacturers or batches. Another angle of looking at this issue is the fact that biological component variation may links with chemical compounds. Yet due to the current lack of understanding of such link, the integration approach is still warranted.

Additionally, we need to emphasize that classification of samples based on selected features would always out-perform those based on all features. Just by selection of 10 features (5 chemical and 5 biological), we could already achieve high accuracies for LDW sample classification. This is largely due to the fact that by selecting features, we could avoid noises that exist in all biological and chemical features.

Moreover, since biological ingredient analysis is an inalienable part of quality evaluation of TCM preparations and possess several advantages that chemical method cannot reach, or namely complementarity, we should not ignore it due to the current deficiency. Although the results in this work were less than perfect, the results of the integration approach had already shown its high accuracy for differentiating samples according to their manufacturers. And it is anticipated that as the methods for biological ingredient analysis are more and more accurate and accustomed, the integrated methods would gain better accuracies.

## Conclusion

In this work, an integrated method based on random forest approach has been proposed, that complement chemical ingredients by biological ingredients, for the assessment of the quality of TCM preparations in a holistic manner. It is a proof-of-concept method that is consisted of two-steps: firstly, selection of representative features from both chemical and biological profiles, and secondly combination and normalization of these representative features.

The advantages of the integrated method could be abstracted as follows: Firstly, chemical and biological ingredients for a TCM preparation have been evaluated simultaneously in one round of experiment. Secondly, based on LDW preparations, we have proven that results based on selected features would perform well, and in most cases better than those based on full sets of chemical or biological features. It should also be noticed that representative features should be selected specifically for every different TCM preparations. Thirdly, the integrated approach could outperform that based on chemical ingredient only approach in many but not all cases.

Both random forest approach and KNN approach have confirmed the two key findings of this work: (1) Biological ingredient could complement chemical ingredient in separating TCM preparation from different manufacturers and batches with high accuracy; (2) classification of samples based on selected features would always out-perform those based on all features (either chemical or biological or both).

We foresee that with the advancement of such integrated approach, more TCM preparations, including TCM preparation with single herb, as well as TCM preparation with the same herb but different sources, would be scrutinized for detailed biological and chemical ingredient profiling and quality assessment. In the future TCM preparation assessment process, two key components would need to be taken care of: a curated database of TCM preparation profiles (biological and chemical), and an improved integration method. Firstly, a large curated TCM preparation database could provide rich information about biological and chemical profiles for these diverse categories of TCM preparations. Secondly, the integrated method could be used in conjugation with such a carefully selected and well curated database of TCM preparation profiles (biological and chemical), so that TCM preparations to be assessed can be compared with this database, and then the quality of the TCM preparation could be described quantitative. As such database become larger with more samples collected, this sample-to-sample comparison could even be extended to a sample-free model, against which the to-be-assessed TCM preparation can be directly compared.

## Supplementary information

Additional file

## Data Availability

The datasets generated and/or analysed during the current study are available in the NCBI Sequence Read Archive (SRA) repository with accession number SRR1049940, https://www.ncbi.nlm.nih.gov/sra/?term=SRR1049940.
