# Supplementary material for: Biological ingredient complement chemical ingredient in the assessment of the quality of TCM preparations
Source: Sci Rep. 2019 Apr 10;9:5853. doi: 10.1038/s41598-019-42341-4 (PMC6458136; doi:10.1038/s41598-019-42341-4)
Supplement: Supplementary file 1 — Additional file [file 41598_2019_42341_MOESM1_ESM.docx]

**Supplementary Information**

**Biological ingredient complement chemical ingredient in the assessment of the quality of TCM preparations**

*Hong Bai ^1,#^, Xianhong Li ^2^, Hongjun Li ^1^, Jialiang Yang ^3,*^,* *Kang Ning ^1,*^*

1. *School of Life Science and Technology, Huazhong University of Science and Technology, Wuhan, Hubei, 430074, China*

*2. College of Biological Sciences, Zhejiang University of Science and Technology, Hangzhou 454000, P. R. China*

*3. Department of Genetics and Genomic Sciences, Icahn School of Medicine at Mount Sinai, NewYork, USA*

^*^ Corresponding author. Tel: +1 917 561 6202, email: [jialiang.yang@mssm.edu](mailto:jialiang.yang@mssm.edu) (Jialiang Yang); Tel: +86 27 87793041, e-mail: ningkang@hust.edu.cn (Kang Ning).

**Contents**

1. Supplementary Table S1. Basic information for 27 commercial LDW samples….………………………………………………………………………S1
2. Supplementary Table S2. RT and PA of 27 LDW samples based on HPLC fingerprint.………………………………………………………………………S2
3. Supplementary Table S3. The species and the relative abundance of detectable species based on ITS2 of 27 LDW samples…………………………………S3
4. Supplementary Table S4. Pair-wise similarities between LDW samples based on HPLC fingerprint………………………………………………………………S4
5. Supplementary Table S5. Pair-wise Euclidean distances between LDW samples based on the relative abundance of the detectable species………………………S5
6. Supplementary Figure S1. HPLC chromatograms of (a) samples S1–S27 at 65 min; (b) samples S1–S27 at 60 min.……………………………………………S6
7. Supplementary text. Abbreviation list............................................................S7

**Supplementary Table S1. Basic information for 27 commercial LDW samples**

**Supplementary Table S2. RT and PA of 27 LDW samples based on HPLC fingerprint**

**Supplementary Table S3. The species and the relative abundance of detectable species based on ITS2 of 27 LDW samples**

**Supplementary Table S4. Pair-wise similarities between LDW samples based on HPLC fingerprint**

**Supplementary Table S5. Pair-wise Euclidean distances between LDW samples based on the relative abundance of the detectable species**

**Figure S1. HPLC chromatograms of (a) samples S1–S27 at 65 min; (b) samples S1–S27 at 60 min**

**List of abbreviations**

**TCM**: Traditional Chinese Medicine

**Ch. P.**: Chinese Pharmacopoeia

**TLC**: Thin Layer Chromatography

**HPLC**: High Performance Liquid Chromatography

**LDW**: Liuwei Dihuang Wan

**HTS**: High-throughput sequencing

**ITS2:** Internal transcribed spacer 2

**RSD**: Relative standard deviation

**RT**: Retention time

**PA**: Peak area

**SFDA**: China’s State Food and Drug Administration

**MCF**: Mean Chromatographic Fingerprint
